# Colonoscopy Indication Algorithm Performance Across Diverse Health Care Systems in the PROSPR Consortium

**DOI:** 10.5334/egems.296

**Published:** 2019-08-02

**Authors:** Andrea N. Burnett-Hartman, Aruna Kamineni, Douglas A. Corley, Amit G. Singal, Ethan A. Halm, Carolyn M. Rutter, Jessica Chubak, Jeffrey K. Lee, Chyke A. Doubeni, John M. Inadomi, V. Paul Doria-Rose, Yingye Zheng

**Affiliations:** 1 Institute for Health Research, Kaiser Permanente Colorado, Denver, CO, US; 2 Fred Hutchinson Cancer Research Center, Seattle, WA, US; 3 Kaiser Permanente Washington Health Research Institute, Seattle, WA, US; 4 Division of Research, Kaiser Permanente Northern California, Oakland, CA, US; 5 Department of Internal Medicine, University of Texas Southwestern Medical Center, Dallas, TX, US; 6 Harold C. Simmons Comprehensive Cancer Center, Dallas, TX, US; 7 RAND Corporation, Santa Monica, California, US; 8 Center for Health Equity and Community Engagement Research, Rochester, MN, US; 9 Department of Family Medicine, Mayo Clinic, Rochester, MN, US; 10 Division of Gastroenterology, University of Washington, School of Medicine, Seattle, WA, US; 11 Division of Cancer Control and Population Sciences, National Cancer Institute, Rockville, Maryland, US

**Keywords:** Colonoscopy, Indication, Colorectal Cancer Screening, Algorithm, Electronic Health Records

## Abstract

**Background:**

Despite the importance of characterizing colonoscopy indication for quality monitoring and cancer screening program evaluation, there is no standard approach to documenting colonoscopy indication in medical records.

**Methods:**

We applied two algorithms in three health care systems to assign colonoscopy indication to persons 50–89 years old who received a colonoscopy during 2010–2013. Both algorithms used standard procedure, diagnostic, and laboratory codes. One algorithm, the KPNC algorithm, used a hierarchical approach to classify exam indication into: diagnostic, surveillance, or screening; whereas the other, the SEARCH algorithm, used a logistic regression-based algorithm to provide the probability that colonoscopy was performed for screening. Gold standard assessment of indication was from medical records abstraction.

**Results:**

There were 1,796 colonoscopy exams included in analyses; age and racial/ethnic distributions of participants differed across health care systems. The KPNC algorithm’s sensitivities and specificities for screening indication ranged from 0.78–0.82 and 0.78–0.91, respectively; sensitivities and specificities for diagnostic indication ranged from 0.78–0.89 and 0.74–0.82, respectively. The KPNC algorithm had poor sensitivities (ranging from 0.11–0.67) and high specificities for surveillance exams. The Area Under the Curve (AUC) of the SEARCH algorithm for screening indication ranged from 0.76–0.84 across health care systems. For screening indication, the KPNC algorithm obtained higher specificities than the SEARCH algorithm at the same sensitivity.

**Conclusion:**

Despite standardized implementation of these indication algorithms across three health care systems, the capture of colonoscopy indication data was imperfect. Thus, we recommend that standard, systematic documentation of colonoscopy indication should be added to medical records to ensure efficient and accurate data capture.

## Introduction

Colorectal cancer (CRC) screening via colonoscopy is associated with decreases in the incidence and mortality of CRC [1,2,3,4], and it is the most frequently used CRC screening procedure in the United States [5]. Colonoscopy is also commonly used for diagnostic reasons in patients with gastrointestinal-related symptoms, such as iron-deficiency anemia [6], and for CRC surveillance in patients at elevated risk due to personal history of CRC or colorectal adenomas [7]. Diagnostic and surveillance colonoscopies tend to have different CRC and adenoma yields [8,9] and greater risks of adverse events [10,11], than screening colonoscopies. Also, because the vast majority of individuals diagnosed with CRC after presentation with symptoms undergo a colonoscopy as part of their diagnostic process, failure to accurately determine exam indication can lead to biased estimates of the benefit of colonoscopy [12,13]. Thus, characterizing colonoscopy indication is important to obtain valid estimates of the population-level risks and benefits of colonoscopy CRC screening [14,15].

Assigning correct indication for colonoscopy may also be important for calculating CRC screening quality metrics. In particular, endoscopist adenoma detection rate (ADR), defined as the percent of screening colonoscopies that have at least one adenoma detected, is used to monitor the quality of CRC screening programs [16]. When calculating ADR as a CRC screening quality metric it is frequently recommended to consider only screening colonoscopies, because colonoscopies performed for other indications tend to have different average ADRs than screening exams and may artificially skew screening exam ADRs [9,17].

Despite the importance of characterizing colonoscopy indication for quality monitoring and cancer screening program evaluation, it is difficult to automate the capture of colonoscopy indication information from electronic health records (EHRs). Colonoscopy indication is often recorded as unstructured text within patient referrals, pre-procedure notes, or endoscopy reports, but standardized, structured data for colonoscopy indication are not available within most health care systems [18]. Thus, the documentation of colonoscopy indication in EHRs may vary between providers and between health care systems.

Furthermore, indication for a given colonoscopy may be noted in multiple sources within the EHR, and these sources may contradict one another [18]. For example, the patient referral may indicate routine CRC screening, but the pre-procedure note or procedure report may list patient symptoms, such as rectal pain, that are indicative of a diagnostic exam. Lab test results, such as results for iron-deficiency anemia tests, also provide information about indication for colonoscopy. Thus, if data from the referral, pre-procedure note, procedure report, and lab results were pooled to obtain a more complete picture of the patient’s medical history, a more comprehensive view for colonoscopy indication may be achieved [19].

To overcome the challenge of obtaining information on colonoscopy indication across multiple providers and sources within the EHR, research teams have developed empirical and data-driven algorithms to assign colonoscopy indication [20,21,22,23,24,25,26]. These algorithms have generally only been tested within the same health care system in which the algorithm was developed [22,23,24,25]. In order to conduct valid, multi-institution CRC screening research, it is important to test the performance of colonoscopy indication algorithms across health care systems. Thus, the objective of this study is to test the generalizability and performance of two algorithms for assigning colonoscopy indication [20,21] across three health care systems within the National Cancer Institute-funded Population-Based Research Optimizing Screening through Personalized Regimens (PROSPR) consortium.

## Methods

### Study Setting

This study was conducted as part of the NCI-funded consortium, Population-based Research Optimizing Screening through Personalized Regimens (PROSPR). The overall aim of PROSPR is to conduct multi-site, coordinated, transdisciplinary research to evaluate and improve cancer screening processes. The ten PROSPR Research Centers reflect the diversity of US delivery system organizations. The PROSPR CRC Research Centers include: Kaiser Permanente Washington (KPWA, formerly known as Group Health), Kaiser Permanente Northern California and Kaiser Permanente Southern California (KPNC/SC), and the Parkland Health & Hospital System/University of Texas Southwestern Medical Center (Parkland-UTSW) [27]. For KPWA and KPNC/SC, eligible patients included health plan members ages 50–89 years old. Those at Parkland-UTSW were patients ages 50–64 years old in this county-based, safety-net health care system [27]. Patients with a history of colectomy or CRC were ineligible for entry in to the PROSPR CRC cohort, but cohort members who developed incident CRC after cohort entry were included. All study activities were approved by the Institutional Review Boards at each Research Center and at the Fred Hutchinson Cancer Research Center, which manages PROSPR data as the Statistical Coordinating Center.

### Sample selection

Among PROSPR CRC cohort members who received a colonoscopy from January 1, 2010 to December 31, 2013, we selected a simple random sample of 600 colonoscopies at each CRC Research Center for manual abstraction of colonoscopy indication and other information from the EHR. This sample size was selected to allow for precise sensitivity and specificity estimates for the two algorithms at each CRC Research Center. We excluded patients with <1 year of prior health plan enrollment, because specific algorithm components required a 1-year look-back period. Additionally, we excluded colonoscopies that did not have sufficient medical records data to identify a gold standard indication based on abstraction.

### Indication algorithms

We applied two separate algorithms to assign colonoscopy indication [20,21]. Both algorithms used utilization and EHR data, including: International Classification of Disease 9^th^ edition (ICD-9) procedure and diagnostic codes, Current Procedural Terminology (CPT) codes, Healthcare Common Procedure Coding System (HCPCS) codes, International Classification of Disease Oncology (ICD-O) codes, and lab codes. The KPNC algorithm was originally developed and validated within Kaiser Permanente Northern California, and it used an approach that categorized each colonoscopy as diagnostic, surveillance, or screening based on the presence or absence of specific diagnostic, procedure, and lab codes. If multiple indications were identified for a single exam, a hierarchical approach was applied in which diagnostic indication took precedence over surveillance, and surveillance took precedence over screening [21]. We modified the KPNC algorithm in order to apply it across all three CRC Research Centers with a standard set of codes and definitions across each research site (see Appendix 1 for codes and definitions used in the present study).

The SEARCH algorithm was developed as part of a large cancer screening research project, the Cancer Screening Effectiveness and Research in Community-based Healthcare Study (SEARCH) [20]. The development of the SEARCH algorithm included analyzing data from colonoscopies with a known indication based on medical record abstraction using a multivariable logistic regression model, estimated using the Least Absolute Shrinkage and Selection Operator (LASSO) algorithm to select prediction variables. The SEARCH algorithm uses the logistic regression model to combine predictors with their estimated coefficients to calculate the probability that an exam was performed to screen for CRC (i.e., screening vs. diagnostic or surveillance exams) [20]. See Appendix 2 for the variables and coefficients used in this algorithm across all CRC Research Centers.

### Standardized gold standard abstraction

At each study site, medical record abstractors used a standardized DatStat instrument to capture patient medical and family history according to the referral for colonoscopy, clinic notes from the visit that prompted the referral, colonoscopy procedure report, and pre-procedure notes. This instrument was adapted from prior, validated colonoscopy indication instruments [18,19,20]. All relevant signs and symptoms that were noted in these sources were recorded in the DatStat instrument (see abstraction variables and instructions in Appendix 3). Abstractors at all CRC Research Centers were trained using a standard abstraction protocol [4], and inter-rater reliability of abstractors within Research Centers was assessed via duplicate abstraction of a 10 percent sample of colonoscopies.

### Gold standard indication assignment

As noted above, indication for colonoscopy can be assessed from various sources [15,18]. For our primary analyses, we used a comprehensive assessment of indication as the gold standard. This gold standard assessment combined abstracted information from the referral, clinic note from the visit that prompted the referral, pre-procedure note, procedure report, and electronically-captured data on laboratory evidence for iron-deficiency anemia in the 180 days prior to the colonoscopy exam or the presence of a positive FIT or FOBT exam in the 365 days prior to the colonoscopy exam. For the gold standard assessment, gastrointestinal-related signs or symptoms noted in any of the above sources, iron deficiency anemia, or a positive FIT or FOBT exam were diagnostic indications. If no diagnostic indications were present, history of inflammatory bowel disease (IBD), CRC, colorectal polyps, or CRC-related genetic syndromes were surveillance indications. If no diagnostic or surveillance indications were present, the indication was screening. If the indication for the colonoscopy was a prior incomplete exam, the indication from the prior exam that was incomplete was used. See Appendix 4 for the decision tree used to assign the gold standard indication for colonoscopy.

### Analysis

The SEARCH algorithm results in an estimated probability that the colonoscopy was performed for screening, which theoretically ranges from 0-1. Depending on the needs of the analysis, a cut-point can be applied to dichotomize the outcome into screening and non-screening indications, or this probability can be used as a continuous variable. The modified KPNC algorithm results in a discreet, categorical exam indication (screening, surveillance, or diagnostic).

The estimated performance of each algorithm is based on the ability to correctly characterize colonoscopy indication according to the gold standard assessment of indication. To evaluate the performance of the SEARCH algorithm to detect screening exams at each CRC Research Center, we used receiver operating characteristics (ROC) curves. We also used the area under the curve (AUC) to summarize the performance of this algorithm to correctly classify screening exams. For the KPNC algorithm, we calculated sensitivity, specificity, and 95 percent confidence intervals for these point estimates for each indication category (screening, surveillance, and diagnostic), stratified by Research Center. To compare the performance of the 2 algorithms for screening indication, we plotted the sensitivity and (1-specificity) for the KPNC algorithm on the same graph as the ROC curve for the SEARCH algorithm. We also compared the specificity of each algorithm and computed 95 percent confidence intervals for these estimates. All analyses were conducted using R version 3.0.2.

In addition to these primary analyses, we conducted exploratory analyses to determine how the source of the indication information from the EHR affects estimates of algorithm performance. For these exploratory analyses, we used only the information from the referral and clinic notes from the visit that prompted the referral to determine the indication according to the referral sources. We also recorded a separate indication based on information from the endoscopy report and pre-endoscopy procedure notes.

## Results

### Study sample characteristics

Characteristics of individuals included in the study sample differed across Research Centers with respect to age, sex, and race/ethnicity (Table 1). Most of those sampled from the KPWA and KPNC/SC cohorts were ≥60 years old; whereas most (66 percent) of the Parkland-UTSW study sample was <60 years old. KPWA and KPNC/SC had slightly more females than males, and about 66 percent of the Parkland-UTSW study sample was female. The KPWA study sample was predominantly Non-Hispanic White (79 percent); KPNC/SC was 53 percent Non-Hispanic White and also had large proportions of Hispanics (21 percent) and Asian American/Pacific Islanders (16 percent); Parkland-UTSW was primarily Hispanic (42 percent) or Non-Hispanic Black (31 percent). KPWA’s study sample had a larger percent of individuals with a comorbidity score of 0 than either KPNC/SC or Parkland-UTSW. This is partially due to larger proportions of individuals with missing comorbidity score data at both KPNC/SC and Parkland-UTSW.

**Table 1:** Study population characteristics, by PROSPR Research Center.

	KPWA N = 600 N (%)	KPNC/SC N = 600 N (%)	Parkland-UTSW N = 596 N (%)

**Age at colonoscopy, yrs.**			
50–59	231 (38%)	261 (44%)	393 (66%)
60–75	309 (52%)	284 (47%)	203 (34%)
76–89	60 (10%)	55 (9%)	0 (0%)
**Sex**			
Female	332 (55%)	322 (54%)	396 (66%)
Male	268 (45%)	278 (46%)	200 (34%)
**Race**			
Non-Hispanic White	474 (79%)	315 (53%)	103 (17%)
Non-Hispanic Black	25 (4%)	44 (7%)	187 (31%)
Hispanic	28 (5%)	125 (21%)	252 (42%)
Asian/Pacific Islander	33 (6%)	96 (16%)	47 (8%)
Other	40 (7%)	20 (3%)	7 (1%)
**Charlson comorbidity index^a^**			
0	324 (54%)	262 (44%)	156 (26%)
1–2	158 (26%)	166 (27%)	138 (23%)
3+	78 (14%)	105 (17%)	41 (7%)
Missing^a^	40 (7%)	67 (11%)	261 (44%)

^a^ Comorbidity index was calculated based on comorbidity status during the calendar year of the index colonoscopy. If data are unavailable for any month during the year, then comorbidity index was set to missing.

### Indications and signs and symptoms for colonoscopy

According to the comprehensive gold standard assessment of colonoscopy indication, about half of the exams at KPNC/SC and Parkland-UTSW were performed for diagnostic indications; at KPWA, 39 percent of colonoscopies were diagnostic (Table 2). If only the referral sources or procedure report/pre-procedure note sources were used to assess indication, the percent of exams that were considered diagnostic decreased at each Research Center. The most common diagnostic signs and symptoms were positive FIT/FOBT (11 percent for KPWA, 22 percent for KPNC/SC, and 13 percent for Parkland-UTSW), rectal bleeding (11 percent for KPWA, 16 percent for KPNC/SC, and 18 percent for Parkland-UTSW), and abdominal pain (10 percent for KPWA, 7 percent for KPNC/SC, and 18 percent for Parkland-UTSW) (Table 2).

**Table 2:** Abstracted indications, signs, and symptoms at colonoscopy exam, by PROSPR Research Center and source of indication data.

	KPWA N = 600 N (%)	KPNC/SC N = 600 N (%)	Parkland-UTSW N = 596 N (%)

**Gold Standard^a^**			
Diagnostic	232 (39%)	303 (51%)	312 (52%)
Screening	209 (35%)	190 (32%)	246 (41%)
Surveillance	132 (22%)	106 (18%)	38 (6%)
Missing	23 (4%)		
**Referral Sources^b^**			
Diagnostic	197 (33%)	257 (43%)	247 (41%)
Screening	248 (41%)	235 (39%)	309 (52%)
Surveillance	94 (16%)	77 (13%)	39 (7%)
Missing	58 (10%)	31 (5%)	1 (<1%)
**Procedure Sources^c^**			
Diagnostic	159 (27%)	230 (38%)	234 (39%)
Screening	262 (44%)	237 (40%)	313 (52%)
Surveillance	135 (23%)	129 (22%)	48 (8%)
Missing	43 (7%)	2 (<1%)	1 (<1%)
**Signs and Symptoms^d^**			
Positive FIT/FOBT	63 (11%)	131 (22%)	76 (13%)
Abnormal sigmoidoscopy, barium enema, or imaging exam	10 (2%)	22 (4%)	18 (3%)
Rectal bleeding	63 (11%)	96 (16%)	105 (18%)
Other GI bleeding	9 (2%)	14 (2%)	22 (4%)
Iron-deficiency anemia	35 (6%)	59 (10%)	39 (7%)
Anemia, other or unspecified	18 (3%)	28 (5%)	33 (6%)
Diarrhea, loose or watery stools	48 (8%)	28 (5%)	30 (5%)
Constipation	28 (5%)	17 (3%)	84 (14%)
Change in bowel habits	30 (5%)	8 (1%)	21 (4%)
Irritable Bowel Syndrome (IBS)	2 (<1%)	6 (1%)	3 (<1%)
Abdominal mass	1 (<1%)	0 (0%)	2 (<1%)
Abdominal pain	59 (10%)	41 (7%)	110 (18%)
Rectal pain	2 (<1%)	3 (<1%)	9 (2%)
weight loss	9 (2%)	7 (1%)	13 (2%)
Suspected new colorectal cancer	0 (0%)	0 (0%)	2 (0.34%)
Inflammatory Bowel Disease (IBD)	16 (3%)	9 (2%)	10 (2%)
Colitis other than IBD	7 (1%)	2 (<1%)	4 (<1%)

^a^ Sources include: referral, clinic notes from the visit that prompted the referral, procedure report, pre-procedure notes, electronic data capture for laboratory-confirmed positive FIT/guaiac FOBT or iron-deficiency anemia. ^b^ Sources include: referral or clinic notes from the visit that prompted the referral. ^c^ Sources include: procedure report or pre-procedure notes. ^d^ Signs and symptoms abstracted from any of the following sources: referral, clinic notes from the visit that prompted the referral, procedure report, or pre-procedure notes, or identified through electronic data capture for laboratory-confirmed positive FIT/FOBT or iron-deficiency anemia. Signs and symptoms are not mutually exclusive.

### Algorithm performance for screening colonoscopy indication

**Figure 1:**
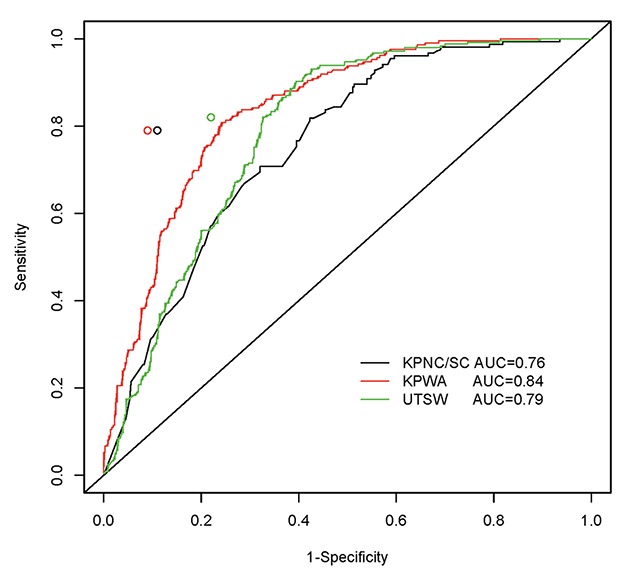
ROC curves and point-estimates for the performance of the SEARCH and KPNC algorithms for detecting screening colonoscopies in each CRC Research Center.

Indication algorithm performance, sensitivity, and specificity for characterizing screening colonoscopy indications are summarized in Table 3, and ROC curve plots are displayed in Figure 1. The overall performance of the SEARCH algorithm for screening indication as measured by the AUC values ranged from 0.76–0.84 across Research Centers (Figure 1 and Table 3). For screening indication, the KPNC algorithm had sensitivities ranging from 0.78–0.82 and specificities ranging from 0.78–0.91 across Research Centers. In comparing the SEARCH and KPNC algorithms by fixing the sensitivity of the SEARCH algorithm to match the sensitivity of the KPNC algorithm, the KPNC algorithm achieved higher specificities than the SEARCH algorithm at each Research Center (0.78–0.91 vs. 0.60–0.76).

**Table 3:** Comparisons of the overall performance, sensitivity, and specificity of the SEARCH and KPNC algorithms for classifying screening colonoscopy exams, by PROPSR Research Center.

	SEARCH Algorithm		KPNC Algorithm	

	AUC^a^	Specificity (95% CI)^b^	Sensitivity (95% CI)	Specificity (95% CI)

**KPWA**	0.84 (0.80, 0.87)	0.76 (0.69, 0.81)	0.79 (0.73, 0.84)	0.91 (0.88, 0.94)
**KPNC/SC**	0.76 (0.71, 0.80)	0.60 (0.53, 0.68)	0.78 (0.73, 0.84)	0.89 (0.85, 0.92)
**Parkland-UTSW**	0.79 (0.75, 0.82)	0.66 (0.60, 0.72)	0.82 (0.77, 0.87)	0.78 (0.74, 0.83)

^a^ Area Under the Curve (AUC) measures the overall performance of the SEARCH indication algorithm for classifying screening colonoscopy exams. ^b^ Sensitivity for the SEARCH algorithm was fixed at the sensitivity estimate from the KPNC indication algorithm and the corresponding specificity estimate for the SEARCH algorithm was reported. Sources include: referral, clinic notes from the visit that prompted the referral, procedure report, pre-procedure notes, electronic data capture for laboratory-confirmed positive FIT/FOBT or iron-deficiency anemia.

### Algorithm performance for diagnostic and surveillance colonoscopy indications

Sensitivities and specificities achieved by the KPNC algorithm for detecting diagnostic exams are summarized in Table 4. Sensitivity for detecting diagnostic exams ranged from 0.78–0.89, and specificity ranged from 0.74–0.82. For detecting surveillance exams, the KPNC algorithm had poor sensitivity, ranging from 0.11–0.67, and high specificity, ranging from 0.95–1.00.

**Table 4:** Sensitivity and specificity of the KPNC algorithm for classifying diagnostic and surveillance colonoscopy exams, by PROPSR Research Center.

	Diagnostic		Surveillance	

	Sensitivity (95% CI)	Specificity (95% CI)	Sensitivity (95% CI)	Specificity (95% CI)

**KPWA**	0.89 (0.85, 0.93)	0.82 (0.78, 0.86)	0.67 (0.59, 0.75)	0.96 (0.94, 0.98)
**KPNC/SC**	0.83 (0.79, 0.87)	0.78 (0.74, 0.83)	0.59 (0.50, 0.69)	0.95 (0.93, 0.97)
**Parkland-UTSW**	0.78 (0.73, 0.82)	0.74 (0.69, 0.79)	0.11 (0.01, 0.20)	1.00 (1.00, 1.00)

Sources include: referral, clinic notes from the visit that prompted the referral, procedure report, pre-procedure notes, electronic data capture for laboratory-confirmed positive FIT/FOBT or iron-deficiency anemia.

## Discussion

Both of the colonoscopy indication algorithms evaluated across CRC Research Centers in the PROSPR Consortium were independently developed in separate health care systems; they used different approaches for algorithm development, but both algorithms relied on a combination of standard procedure, diagnostic, and laboratory codes to predict colonoscopy indication [20,21]. The use of utilization and other standard data that are available across health care systems allowed for the implementation and testing of these algorithms outside of the systems in which each algorithm was developed. Both algorithms had modest performance for detecting screening colonoscopies, but indication for colonoscopy was still misclassified for a portion of procedures; in particular, surveillance indications were not well-captured.

In the present study, the SEARCH algorithm had an estimated AUC ranging from 0.76-0.84 across the three Research Centers. This is lower than the estimated AUC (0.94) when the SEARCH algorithm was applied to the training dataset that was used to develop the algorithm [20]. The lower performance of the SEARCH algorithm in the present analysis may be due in part to additional exclusions applied to the SEARCH training data; in addition to individuals with prior CRC, those with IBD or a strong family history of CRC were excluded; these individuals are more likely to undergo surveillance exams which are difficult to correctly classify using only electronic health data. Thus, the exclusion of individuals whose colonoscopy was more likely to have a surveillance indication may have enriched the training dataset with colonoscopies that the algorithm is better at classifying correctly. In the present study, a random sample of the PROSPR CRC population receiving colonoscopy was selected. Using a random sample of the population receiving colonoscopy provided an estimate of the model performance in each system that is generalizable to colonoscopy recipients in each Research Center’s population.

The KPNC algorithm allowed for the assignment of colonoscopies into one of three categories: screening, surveillance, or diagnostic [21]. In comparing the KPNC algorithm to the SEARCH algorithm, we found that the KPNC algorithm had better performance than the SEARCH algorithm for detecting screening colonoscopies within each Research Center. This was evidenced by the higher specificities achieved by the KPNC algorithm when the sensitivities were equal between the two algorithms. Although the KPNC algorithm performed relatively well for screening and diagnostic indications across Research Centers, the algorithm had wide variability in its sensitivity for surveillance exams. We explored reasons for this by reviewing the abstracted data for surveillance indications and found that this variability was primarily due to the algorithm failing to capture information on prior polyps as an indication for surveillance exams in Research Centers which lacked electronic data on prior polyp diagnosis. Information on prior polyps is often stored as unstructured text in clinic notes or reports, and it may not show up as a diagnosis code in utilization data. Surveillance exams accounted for 6–22 percent of colonoscopies in these Research Centers.

Compared to other indication algorithms, the KPNC algorithm tended to have comparable sensitivity and superior specificity [22,23,24,25], or similar performance, for identifying screening exams. For diagnostic exams, the KPNC algorithm also had similar [26] performance to previously published algorithms. Thus, despite the KPNC algorithm having been developed and tested in one health care system, when tested across three separate systems, it performed comparable to, or better than, other colonoscopy indication algorithms that were developed and tested within a single system. Therefore, the implementation and validation of the KPNC algorithm may be useful across health care systems to obtain an automated and standardized assessment of screening and diagnostic colonoscopy indications.

While both of the algorithms we examined had good accuracy, neither had perfect or near perfect accuracy for identification of screening colonoscopies. These algorithms provide a useful approximation of colonoscopy indication, but appropriate statistical methods are needed to account for their imprecision [14,28]. This is also true of the KPNC algorithm when identifying diagnostic colonoscopies.

In addition to testing indication algorithm performance across multiple health care systems, we conducted exploratory analyses to evaluate how the source of abstracted colonoscopy indication data affected the estimated performance of these algorithms (Supplemental Tables 1–2). This is important, because indication for colonoscopy can be documented in multiple places in the EHR [18]. Thus, we aimed to determine if algorithm performance varied according to which source was used to assign colonoscopy indication. Our results suggested that the algorithms’ overall performance, sensitivity, and specificity varied only slightly according to which source was used to assess colonoscopy indication. This is consistent with prior research showing that referral and endoscopy report assessments of colonoscopy indication have good agreement with combined information from multiple sources in the EHR [29,30].

One of the strengths of this study is the inclusion of a large, random sample of patients undergoing colonoscopy at each Research Center. This allowed for robust estimates of algorithm performance that are generalizable to the population within each Center. It also allowed for precise calculations of the error in each algorithm’s assignment of colonoscopy indication and in each Research Center. This is important because statistical methods are available to adjust for differences in indication algorithm performance across sites using estimates from our analyses [14]. These methods are rarely applied, in part due to the lack of data on algorithm performance. Another strength is that the algorithms were tested across diverse health care systems, including systems other than those in which the algorithms were developed. We also evaluated model performance using multiple sources of data on indication from the EHR and applied the same gold standard assessment of colonoscopy indication across all Research Centers.

Despite these strengths, there were several limitations. One limitation is that these algorithms were tested in relatively closed health care systems where most of the care that a patient receives is done within a single health care system or tracked via claims data for care that is performed outside of the health care system. Thus, these algorithms may not perform well in open health care models where patients receive care from multiple systems and do not have a single EHR or claims database that tracks patient care. Additional research to determine the performance of these algorithms in open health care models is needed. Another limitation is that the gold standard assessment for colonoscopy indication was limited to the sources within the EHR that are most likely to contain information on colonoscopy indication. However, it is possible that colonoscopy indication, such as prior history of colorectal polyps, may have been noted in the clinic notes for a visit that was not reviewed. Despite this limitation, the EHR sources that we abstracted for the gold standard assessment of indication were validated sources for colonoscopy indication that have been used in prior studies [18,19,20].

## Conclusion

Our study illustrates that when an algorithm is transferred to an outside system, it is valuable to conduct a validity study to understand the performance of the algorithm and to estimate the parameters needed to statistically adjust for the imprecision of the algorithm. Despite standardized implementation of these indication algorithms across the health care systems comprising the PROSPR CRC Research Centers, the capture of colonoscopy indication data was imperfect. Thus, we recommend that national CRC screening guidelines promote consistent and systematic documentation of colonoscopy indication in a structured manner. This includes providing standard definitions for screening, surveillance, and diagnostic exams, and advocating for a unique field within the EHR with separate codes to distinguish colonoscopy indications. With the increasing prevalence of electronic endoscopic reporting systems, it is becoming more feasible to require standardized documentation and reporting of colonoscopy indication. Thus, endoscopists should be incentivized to accurately document the colonoscopy indication to allow electronic/automated extraction of indication from the EHR.

## Additional Files

The additional files for this article can be found as follows:

10.5334/egems.296.s1 Appendix 1. KPNC Algorithm Logic and Codes.

10.5334/egems.296.s2 Appendix 2. SEARCH Indication Algorithm Instructions and Codes.

10.5334/egems.296.s3 Appendix 3. PROSPR Colonoscopy Indication Gold Standard Abstraction Variables and Abstractor Instructions.

10.5334/egems.296.s4 Appendix 4. Decision Tree for Gold Standard Assessment of Indication Based on Electronic Lab Data and Medical Record Abstraction.

10.5334/egems.296.s5 Supplemental Table 1. Comparisons of the overall performance, sensitivity, and specificity of the SEARCH and KPNC algorithms for classifying screening colonoscopy exams, by PROPSR Research Center and source of indication assessment.

10.5334/egems.296.s6 Supplemental Table 2. Sensitivity and specificity of the KPNC algorithm for classifying diagnostic and surveillance colonoscopy exams, by PROPSR Research Center and source of indication assessment.
