# Supplementary material for: Colonoscopy Indication Algorithm Performance Across Diverse Health Care Systems in the PROSPR Consortium
Source: EGEMS (Wash DC). 2019 Aug 2;7(1):37. doi: 10.5334/egems.296 (PMC6676916; doi:10.5334/egems.296)
Supplement: Appendix 1. — KPNC Algorithm Logic and Codes. [file egems-7-1-296-s1.pdf]

Appendix 1: KPNC Algorithm Logic and Codes

**1**	**Any History of Colectomy? (If yes, indication = diagnostic)**	
	**Colectomy CPT 4 procedure codes**	
	44150	Total colectomy
	44151	Total colectomy
	44152	Total colectomy
	44153	Total colectomy
	44155	Total colectomy
	44156	Total colectomy
	44157	Total colectomy
	44158	Total colectomy
	44210	Total colectomy
	44211	Total colectomy
	44212	Total colectomy
	44140-7, 44160,44204-207,44213,44227	Partial colectomy
	45110-4, 45116, 45119, 45123, 45126, 45160, 45170	Rectum (Surgery)
	44144, 44310, 44312, 44314, 44316	Ileostomy
	**Colectomy ICD 9 diagnosis codes**	
	V45.72	HX of Total colectomy
	**Colectomy ICD 9 procedure codes**	
	45.8	Total intra-abdominal colectomy
	45.7	Open and other partial excision of large intestine
**2**	**Any History of IBD? (If yes, indication = diagnostic)**	
	**IBD ICD 9 diagnosis codes**	
	555	Regional enteritis
	556	Ulcerative colitis
	557	Vascular insufficiency of intestine
	558	Other and unspecified noninfectious gastroenteritis and colitis
**3**	**Positive FOBT/FIT in ≤ 365 days before current exam? (If yes, indication = diagnostic)**	
	**FOBT KP lab codes**	
	3502000	Occult Blood, Feces Single
	3502010	Occult Blood #1, Feces
	3502020	Occult Blood #2, Feces
	3502030	Occult Blood #3, Feces
	3502040	Occult Blood Series 3, Feces

	3502110	Occult Blood #4, Feces
	3502120	Occult Blood #5, Feces
	3502130	Occult Blood #6, Feces
	3502140	Occult Blood Series 6, Feces
	3506000	Combo Fecal Occult Blood (REG)
	3506010	Hemoccult Sensa
	3506025	Combination Fecal Occult Blood
	3506050	Fecal Hemoglobin
	9407015	Fecal Globin
	9523250	Fecal Globin
**4**	**The gastroenterology referral or databases include any of the following ≤ 180 days before current exam (not including the procedure date): abdominal pain; iron-deficiency anemia; gastrointestinal bleeding or blood in stools; diarrhea, constipation, or change in bowel habits; unexplained weight loss; diverticulitis? (If yes, indication = diagnostic)**	
	**GI-related symptoms ICD 9 diagnosis codes**	**Abdominal pain**
	789.0	Abdominal pain
		**Diarrhea**
	564.5	Functional diarrhea
	787.91	Diarrhea
		**Constipation**
	564.0	Constipation
		**Diverticulitis**
	562.11	Diverticulitis of colon without mention of hemorrhage
	562.13	Diverticulitis of colon with hemorrhage
		**Anemia**
	280.8	Other specified iron deficiency anemias
	280.9	Iron deficiency anemia, unspecified
	**KP Lab codes**	
	0800325	Iron
	0800830	Iron & TIBC
	0800835	Transferrin Saturation (REG)
	0803019	Beta-2 Transferrin, Body Fluid
	0803019	Beta-2 Transferrin, CSF
	1000820	Ferritin (REG)
	1001110	Unsaturated Iron Binding Cap.
	1001114	Transferrin % Saturation
	1001115	IRON + TIBC (REG)
	1001125	Total Iron Binding Capacity
	1001245	Iron
	1002840	Transferrin

9002520	Transferrin (NIC)
9014050	SK Iron Panel (Smith/Kline)
9400746	Transferrin
	**weight loss**
783.2	Abnormal loss of weight and underweight
	**blood in stool**
578.1	Blood in stool
	**GI bleeding**
531.0	Gastric ulcer, Acute with hemorrhage
531.2	Gastric ulcer, Acute with hemorrhage and perforation
531.4	Gastric ulcer, Chronic or unspecified with hemorrhage
531.6	Gastric ulcer, Chronic or unspecified with hemorrhage and perforation
532.0	Duodenal ulcer, Acute with hemorrhage
532.2	Duodenal ulcer, Acute with hemorrhage and perforation
532.4	Duodenal ulcer, Chronic or unspecified with hemorrhage
532.6	Duodenal ulcer, Chronic or unspecified with hemorrhage and perforation
533.0	Peptic ulcer (site unspecified), Acute with hemorrhage
533.2	Peptic ulcer (site unspecified), Acute with hemorrhage and perforation
533.4	Peptic ulcer (site unspecified), Chronic or unspecified with hemorrhage
533.6	Peptic ulcer (site unspecified), Chronic or unspecified with hemorrhage and perforation
534.0	Gastrojejunal ulcer, Acute with hemorrhage
534.2	Gastrojejunal ulcer, Acute with hemorrhage and perforation
534.4	Gastrojejunal ulcer, Chronic or unspecified with hemorrhage
534.6	Gastrojejunal ulcer, Chronic or unspecified with hemorrhage and perforation
535.11	Atrophic gastritis, with hemorrhage (Atrph gastritis w hmrhg)
535.21	Gastric mucosal hypertrophy, with hemorrhage (Gstr mcsl hyprt w hmrg).
535.31	Alcoholic gastritis, with hemorrhage
535.41	Other specified gastritis, with hemorrhage

	535.51	Unspecified gastritis and gastroduodenitis, with hemorrhage
	535.61	Duodenitis, with hemorrhage
	562.02	Diverticulosis of small intestine with hemorrhage
	562.03	Diverticulitis of small intestine with hemorrhage
	562.12	Diverticulosis of colon with hemorrhage
	562.13	Diverticulitis of colon with hemorrhage
	569.3	Hemorrhage of rectum and anus
	578	Gastrointestinal hemorrhage
**5**	**Initial CRC diagnostic code by pathology or cancer registry is ≤ 180 days before current exam? (If yes, indication = diagnostic)**	
	**CRC ICD-O Codes**	
	C18.0	Malignant neoplasm cecum
	C18.1	Malignant neo appendix
	C18.2	Malignant neo ascend colon
	C18.3	Malignant neo hepatic flexure
	C18.4	Malignant neo transverse colon
	C18.5	Malignant neo splenic flexure
	C18.6	Malignant neo descend colon
	C18.7	Malignant neo sigmoid colon
	C18.8	Malignant neo colon NEC/Overlapping
	C18.9	Malignant neo colon NOS
	C19.9	Malignant neo rectosigmoid jct
	C20.9	Malignant neopl rectum
	**CRC ICD 9 codes**	
	153	Malignant neoplasm of colon
	153.0	Malignant neo hepatic flexure
	153.1	Malignant neo transverse colon
	153.2	Malignant neo descend colon
	153.3	Malignant neo sigmoid colon
	153.4	Malignant neoplasm cecum
	153.5	Malignant neo appendix
	153.6	Malignant neo ascend colon
	153.7	Malignant neo splenic flexure
	153.8	Malignant neo colon NEC/Overlapping
	153.9	Malignant neo colon NOS
	154	
	154.0	Malignant neoplasm rectosigmoid jct
	154.1	Malignant neoplasm rectum
	197.5	
	209.1	

	230.3	Carcinoma in situ of colon
	230.4	Carcinoma in situ of rectum
**6**	**Had a sigmoidoscopy or colonoscopy with an ICD-9 diagnosis of polyp ≤ 365 days before current exam? (If yes, indication = diagnostic)**	
	**ICD 9 diagnosis codes**	**colon/rectal adenoma or polyp**
	235.2	Neoplasm of uncertain behavior of stomach, intestines, and rectum
	209.5	Benign carcinoid tumors of the appendix, large intestine, and rectum
	211.3	Benign neoplasm of colon
	211.4	Benign neoplasm of rectum and anal canal
	569.0	Anal and rectal polyp
		**colon/rectal mass**
	569.9	Unspecified disorder of intestine
	787.9	Other symptoms involving digestive system
**7**	**Initial CRC diagnostic code by pathology or cancer registry is > 180 days before current exam? (If yes, indication = surveillance)**	
	**CRC ICD-O Codes**	
	C18.0	Malignant neoplasm cecum
	C18.1	Malignant neo appendix
	C18.2	Malignant neo ascend colon
	C18.3	Malignant neo hepatic flexure
	C18.4	Malignant neo transverse colon
	C18.5	Malignant neo splenic flexure
	C18.6	Malignant neo descend colon
	C18.7	Malignant neo sigmoid colon
	C18.8	Malignant neo colon NEC/Overlapping
	C18.9	Malignant neo colon NOS
	C19.9	Malignant neo rectosigmoid jct
	C20.9	Malignant neopl rectum
	**CRC ICD 9 codes**	
	153	Malignant neoplasm of colon
	153.0	Malignant neo hepatic flexure
	153.1	Malignant neo transverse colon
	153.2	Malignant neo descend colon
	153.3	Malignant neo sigmoid colon
	153.4	Malignant neoplasm cecum
	153.5	Malignant neo appendix

	153.6	Malignant neo ascend colon
	153.7	Mal neo splenic flexure
	153.8	Malignant neo colon NEC/Overlapping
	153.9	Malignant neo colon NOS
	154	Malignant neoplasm of rectum rectosigmoid junction and anus
	154.0	Mal neo rectosigmoid jct
	154.1	Malignant neoplasm rectum
	197.5	Secondary malignant neoplasm of large intestine and rectum
	209.1	
		Carcinoma in situ of colon
	230.3	
	230.4	Carcinoma in situ of rectum
**8**	**Only has ICD diagnosis of CRC/history of CRC? (If yes, indication = surveillance)**	
	**V-codes and ICD-9 codes for prior CRC**	
		**colon cancer personal history**
	V10.05	Personal hx of large intestine cancer
	V10.06	Personal hx of cancer of the rectum, rectosigmoid junction, or anus
**9**	**Most recent diagnostic code for polyp is > 365 days before current exam? (If yes, indication = surveillance)**	
	**ICD 9 diagnosis codes**	
	235.2	
		**colon/rectal polyp**
	209.5	
		Benign neoplasm of colon
	211.3	
	211.4	Benign neoplasm of rectum and anal canal
	569.0	Anal and rectal polyp
		**colon/rectal mass**
	569.9	Unspecified disorder of intestine
	787.9	Other symptoms involving digestive system
**10**	**Only has prior ICD diagnosis of history of colon polyps? (If yes, indication = surveillance)**	
	**V-code for prior polyps**	
		**colon polyp personal history**
	V12.72	Colonic polyps

**11**	**If "No" to all decision nodes above, does the participant have ≥365 days continuous health system enrollment prior to colonoscopy (allowing for up to a 90-day gap in enrollment)? (If yes, indication =screening; if no, indication= unknown)**
