# Supplementary material for: Colonoscopy Indication Algorithm Performance Across Diverse Health Care Systems in the PROSPR Consortium
Source: EGEMS (Wash DC). 2019 Aug 2;7(1):37. doi: 10.5334/egems.296 (PMC6676916; doi:10.5334/egems.296)
Supplement: Appendix 2. — SEARCH Indication Algorithm Instructions and Codes. [file egems-7-1-296-s2.pdf]

Appendix 2: SEARCH Indication Algorithm Instructions and Codes

Note: To implement the SEARCH algorithm, create dichotomous Yes/No (0/1) variables using the look-back periods and codes for each symptom/sign below. The look-back period starts with the day of colonoscopy being day 0 and going backwards in time. Some look-back periods start with day 1, and these correspond to the look-back period starting the day prior to colonoscopy. If at the time of colonoscopy, the participant does not have at least 365 days of continuous prior health system coverage starting at day 0 and going back 365 days (allowing for a 90-day gap in prior coverage), the SEARCH indication variable should be marked as “Unknown”.

Predictor	Look-back period (days)	Code or code grouping used by KPWA	Coefficient value
(Intercept)			1.648517
Age Squared	0		-0.000464
Iron deficiency anemia	0-365	280.0, 280.9	-0.919223
Functional digestive disorder	0-365	564.0, 564.00, 564.09, 564.1, 564.7, 564.81, 564.89, 564.9	-0.865357
Rectal bleeding, Hemorrhage, BRBPR	0-365	569.3	-1.343965
GI bleed, stool (4)[a]	0-365	578, 578.1, 578.9, 792.1	-1.167781
Abdominal distension	0-365	787.3	-0.733454
Abdominal pain	0-365	789.0, 789.00, 789.01, 789.02, 789.03, 789.04, 789.05, 789.06, 789.07, 789.09	-0.184290
Nausea/vomiting	1-365	787.0, 787.01, 787.02, 787.03, 787.04	-0.055797
Nausea/vomiting	0-365	787.0, 787.01, 787.02, 787.03, 787.04	-0.649945
Rectal polyp	1-365	569.0	-1.819016

Note: Only the codes listed in this table should be used for creating the dichotomous variables for the SEARCH indication algorithm. Do not substitute other codes.

[a]There were multiple variables for this sign/symptom in the SEARCH model used for selecting the best variables for prediction. The one included in this table is the variable selected for the final model. The variable number (i.e. “(4)”) was retained for easy reference back to the SEARCH indication manuscript.

Classification of screening colonoscopy using dichotomized predicted probabilities

For each colonoscopy, the predicted probability of screening indication is estimated using the linear combination of the regression model coefficients and the subject’s covariate values from the table above. The estimated probability that a colonoscopy is screening, p, is calculated using a log odds transformation of the linear predictor, Xß.

$$\hat{p} = 1/(1+\exp(-1*X\beta)), \text{ where } X\beta = \beta_0 + \beta_1*\text{predictor1} + \beta_2 * \text{predictor2} + \dots \beta_n*\text{predictor n}$$
