# Supplementary material for: Colonoscopy Indication Algorithm Performance Across Diverse Health Care Systems in the PROSPR Consortium
Source: EGEMS (Wash DC). 2019 Aug 2;7(1):37. doi: 10.5334/egems.296 (PMC6676916; doi:10.5334/egems.296)
Supplement: Appendix 3. — PROSPR Colonoscopy Indication Gold Standard Abstraction Variables and Abstractor Instructions. [file egems-7-1-296-s3.pdf]

Appendix 3: PROSPR Colonoscopy Indication Gold Standard Abstraction Variables and Abstractor Instructions

1. **PROSPR Study ID**
2. **Month/year of colonoscopy**
3. **Age at colonoscopy**
4. **Sex**

5.0 Indication for Colonoscopy - Referral

Abstractor Instructions:

Look for signs, symptoms, or other information that indicates the reason for colonoscopy according to the patient referral or clinic note from the encounter (including in-person visits, phone consults, or emails) that prompted the referral only.
Mark all that apply for the reason for colonoscopy according to the referral and clinic note from the encounter that prompted the referral.

1. Screening
2. Personal history of polyps/adenomas
3. Positive FIT/FOBT
4. Abnormal sigmoidoscopy (e.g. polyp/adenoma found at sigmoidoscopy)
5. Abnormal barium enema or imaging exam (X-ray, CT scan, etc.)
6. Rectal bleeding, bright red blood per rectum, blood on toilet paper, hematochezia (blood observed in the stool or in toilet)
7. Other GI bleeding (upper GI bleeding, melena or black tarry stools)
8. Iron-deficiency anemia
9. Anemia, other types or unspecified type
10. Diarrhea, loose or watery stools
11. Constipation
12. Change in bowel habits
13. Irritable Bowel Syndrome (IBS)
14. Abdominal mass
15. Abdominal pain
16. Rectal pain
17. Unintentional weight loss (includes anorexia)
18. Personal history of colorectal cancer (i.e. colon, rectal, or colorectal)
19. Metastatic cancer work-up
20. Colorectal cancer was suspected
21. Therapeutic or treatment of a condition

22. FOBT/FIT that was collected at home
23. Prior incomplete colonoscopy exam
24. Ordered at the same time as another exam
25. Neither the referral note nor the clinic note from the encounter that prompted the referral was found
26. Inflammatory bowel disease (IBD), including Crohn's or ulcerative colitis (UC)
27. Colitis other than IBD
28. Family history of colorectal cancer
29. Family history of FAP, Lynch (HNPCC), or other genetic syndrome
30. Personal history of FAP, Lynch (HNPCC), or other genetic syndrome
31. Other: Specify___________________
32. Unknown; Reason was not specified or documented

--PAGE BREAK--

Abstractor Instructions: If the reason for colonoscopy was "23. Prior incomplete colonoscopy exam" according to the referral or encounter that prompted the referral <u>and</u> no other reasons are noted in the referral, go to the referral or encounter that prompted the referral for the prior colonoscopy exam that was incomplete. This should be for the colonoscopy procedure directly proceeding the index colonoscopy exam. Enter the date of the prior incomplete colonoscopy (6.0) and abstract the reason for this incomplete colonoscopy (7.0) according to the referral or encounter that prompted the referral for this prior incomplete colonoscopy.

6.0 Date of prior incomplete colonoscopy exam (month/year):

7.0 Indication for Colonoscopy- Referral (Prior Incomplete Colonoscopy Exam)

Abstractor Instructions

<u>Mark all that apply</u> for the indication for colonoscopy according to the referral and clinic note from the encounter that prompted the referral for the prior incomplete colonoscopy.

1. Screening
2. Personal history of polyps/adenomas
3. Positive FIT/FOBT
4. Abnormal sigmoidoscopy (e.g. polyp/adenoma found at sigmoidoscopy)
5. Abnormal barium enema or imaging exam (X-ray, CT scan, etc.)
6. Rectal bleeding, bright red blood per rectum, blood on toilet paper, hematochezia (blood observed in the stool or in

toilet)
7. Other GI bleeding (upper GI bleeding, melena or black tarry stools)
8. Iron-deficiency anemia
9. Anemia, other types or unspecified type
10. Diarrhea, loose or watery stools
11. Constipation
12. Change in bowel habits
13. Irritable Bowel Syndrome (IBS)
14. Abdominal mass
15. Abdominal pain
16. Rectal pain
17. Unintentional weight loss (includes anorexia)
18. Personal history of colorectal cancer (i.e. colon, rectal, or colorectal)
19. Metastatic cancer work-up
20. Colorectal cancer was suspected
21. Therapeutic or treatment of a condition
22. FOBT/FIT that was collected at home
23. Prior incomplete colonoscopy exam
24. Ordered at the same time as another exam
25. Neither the referral note nor the clinic note from the encounter that prompted the referral was found
26. Inflammatory bowel disease (IBD), including Crohn's or ulcerative colitis (UC)
27. Colitis other than IBD
28. Family history of colorectal cancer
29. Family history of FAP, Lynch (HNPCC), or other genetic syndrome
30. Personal history of FAP, Lynch (HNPCC), or other genetic syndrome
31. Other: Specify___________________
32. Unknown; Reason was not specified or documented

--PAGE BREAK--

8.0 Indication for Colonoscopy - Colonoscopy Procedure Report

Abstractor Instructions: Look for signs, symptoms, or other information that indicates the reason for colonoscopy <u>according to the indication or patient history section of the colonoscopy procedure report or same day pre-procedure note only.</u> <u>Mark all that apply</u> for the reason for colonoscopy according to the colonoscopy procedure report or same day pre-procedure note.

1. Screening
2. Personal history of polyps/adenomas

3. Positive FIT/FOBT
4. Abnormal sigmoidoscopy (e.g. polyp/adenoma found at sigmoidoscopy)
5. Abnormal barium enema or imaging exam (X-ray, CT scan, etc.)
6. Rectal bleeding, bright red blood per rectum, blood on toilet paper, hematochezia (blood observed in the stool or in toilet)
7. Other GI bleeding (upper GI bleeding, melena or black tarry stools)
8. Iron-deficiency anemia
9. Anemia, other types or unspecified type
10. Diarrhea, loose or watery stools
11. Constipation
12. Change in bowel habits
13. Irritable Bowel Syndrome (IBS)
14. Abdominal mass
15. Abdominal pain
16. Rectal pain
17. Unintentional weight loss (includes anorexia)
18. Personal history of colorectal cancer (i.e. colon, rectal, or colorectal)
19. Metastatic cancer work-up
20. Colorectal cancer was suspected
21. Therapeutic or treatment of a condition
22. FOBT/FIT that was collected at home
23. Prior incomplete colonoscopy exam
24. Ordered at the same time as another exam
25. Neither the colonoscopy procedure report nor the same-day pre-procedure clinic note was found
26. Inflammatory bowel disease (IBD), including Crohn's or ulcerative colitis (UC)
27. Colitis other than IBD
28. Family history of colorectal cancer
29. Family history of FAP, Lynch (HNPCC), or other genetic syndrome
30. Personal history of FAP, Lynch (HNPCC), or other genetic syndrome
31. Other: Specify___________________
32. Unknown; Reason was not specified or documented

---PAGE BREAK---

Abstractor Instructions: If the reason for colonoscopy was "23. Prior incomplete colonoscopy exam" according to the procedure report or same day pre-procedure clinic note <u>and</u> no other reasons are noted for the present exam, go to the procedure report or same day pre-procedure

clinic note for the prior colonoscopy exam that was incomplete. This should be for the colonoscopy procedure directly proceeding the index colonoscopy exam. Enter the date of the prior incomplete colonoscopy (9.0) and abstract the reason for this incomplete colonoscopy (10.0) according to the procedure report or same day pre-procedure clinic note for this prior incomplete colonoscopy.

9.0 Date of prior incomplete colonoscopy exam (month/year):

10.0 Indication for Colonoscopy- Colonoscopy Procedure Report (Prior Incomplete Colonoscopy Exam)

Abstractor instructions: Mark all that apply for the indication for colonoscopy according to the procedure report and same day pre-procedure note for the prior incomplete colonoscopy.

1. Screening
2. Personal history of polyps/adenomas
3. Positive FIT/FOBT
4. Abnormal sigmoidoscopy (e.g. polyp/adenoma found at sigmoidoscopy)
5. Abnormal barium enema or imaging exam (X-ray, CT scan, etc.)
6. Rectal bleeding, bright red blood per rectum, blood on toilet paper, hematochezia (blood observed in the stool or in toilet)
7. Other GI bleeding (upper GI bleeding, melena or black tarry stools)
8. Iron-deficiency anemia
9. Anemia, other types or unspecified type
10. Diarrhea, loose or watery stools
11. Constipation
12. Change in bowel habits
13. Irritable Bowel Syndrome (IBS)
14. Abdominal mass
15. Abdominal pain
16. Rectal pain
17. Unintentional weight loss (includes anorexia)
18. Personal history of colorectal cancer (i.e. colon, rectal, or colorectal)
19. Metastatic cancer work-up
20. Colorectal cancer was suspected
21. Therapeutic or treatment of a condition
22. FOBT/FIT that was collected at home
23. Prior incomplete colonoscopy exam
24. Ordered at the same time as another exam
25. Neither the colonoscopy procedure report nor the same-day pre-procedure clinic note was found
26. Inflammatory bowel disease (IBD), including Crohn's or ulcerative colitis (UC)
27. Colitis other than IBD

28. Family history of colorectal cancer
29. Family history of FAP, Lynch (HNPCC), or other genetic syndrome
30. Personal history of FAP, Lynch (HNPCC), or other genetic syndrome
31. Other: Specify____________________
32. Unknown; Reason was not specified or documented

--------------------------------------PAGE BREAK--

11.0 Abstraction is complete (Yes, No, Hold to inquiry study team)
