# Supplementary material for: Colonoscopy Indication Algorithm Performance Across Diverse Health Care Systems in the PROSPR Consortium
Source: EGEMS (Wash DC). 2019 Aug 2;7(1):37. doi: 10.5334/egems.296 (PMC6676916; doi:10.5334/egems.296)
Supplement: Appendix 4. — Decision Tree for Gold Standard Assessment of Indication Based on Electronic Lab Data and Medical Record Abstraction. [file egems-7-1-296-s4.pdf]

Appendix 4: Decision Tree for Gold Standard Assessment of Indication Based on Electronic Lab Data and Medical Record Abstraction

Did electronic data identify either of the following?

1. A positive FIT/FOBT in the 365 days prior to the colonoscopy
2. Laboratory evidence of iron-deficiency anemia in the 180 days prior to colonoscopy.

Yes → **Diagnostic**

↓ **No**

Did the patient have any of the follow signs or symptoms?[a]

1. Positive FIT/FOBT
2. Abnormal sigmoidoscopy (e.g. polyp/adenoma found at sigmoidoscopy)
3. Abnormal barium enema or imaging exam (X-ray, CT scan, etc.)
4. Rectal bleeding, bright red blood per rectum, blood on toilet paper, hematochezia (blood observed in the stool or in toilet)
5. Other GI bleeding (upper GI bleeding, melena or black tarry stools)
6. Iron-deficiency anemia
7. Anemia, other types or unspecified type
8. Diarrhea, loose or watery stools
9. Constipation
10. Change in bowel habits
11. Irritable Bowel Syndrome (IBS)
12. Abdominal mass
13. Abdominal pain
14. Rectal pain
15. Unintentional weight loss (includes anorexia)
16. Metastatic cancer work-up
17. Suspected new colorectal cancer
18. Therapeutic or treatment of a condition
19. Colitis other than IBD

Yes → **Diagnostic**

↓ **No**

Does the patient have a personal history of any of the following? [a]

1. Inflammatory bowel disease (IBD), including Crohn's or ulcerative colitis (UC)
2. Personal history of polyps/adenomas
3. Personal history of colorectal cancer (i.e. colon, rectal, or colorectal)
4. Personal history of FAP, Lynch (HNPCC), or other genetic syndrome

Yes → **Surveillance**

↓ **No**

Screening

[a]Sources include: laboratory information on fecal blood tests and iron-deficiency anemia, and abstracted information from the patient referral or clinic notes from the visit that prompted the referral, and procedure report or pre-procedure notes
