# Supplementary material for: Colonoscopy Indication Algorithm Performance Across Diverse Health Care Systems in the PROSPR Consortium
Source: EGEMS (Wash DC). 2019 Aug 2;7(1):37. doi: 10.5334/egems.296 (PMC6676916; doi:10.5334/egems.296)
Supplement: Supplemental Table 1. — Comparisons of the overall performance, sensitivity, and specificity of the SEARCH and KPNC algorithms for classifying screening colonoscopy exams, by PROPSR Research Center and source of indication assessment. [file egems-7-1-296-s5.pdf]

Supplemental Table 1. Comparisons of the overall performance, sensitivity, and specificity of the SEARCH and KPNC algorithms for classifying screening colonoscopy exams, by PROPSR Research Center and source of indication assessment

	SEARCH Algorithm		KPNC Algorithm	
	AUC[a]	Specificity[b]	Sensitivity	Specificity
**KPWA**				
Referral[c]	0.80	0.82	0.67	0.92
Procedure Report/Pre-procedure note[d]	0.78	0.80	0.64	0.93
Gold Standard Sources[e]	0.84	0.78	0.79	0.91
**KPNC/SC**				
Referral[c]	0.73	0.64	0.64	0.90
Procedure Report/Pre-procedure note[d]	0.72	0.62	0.68	0.90
Gold Standard Sources[e]	0.76	0.60	0.78	0.89
**Parkland-UTSW**				
Referral[c]	0.77	0.67	0.78	0.88
Procedure Report/Pre-procedure note[d]	0.80	0.76	0.71	0.81
Gold Standard Sources[e]	0.79	0.66	0.82	0.78

[a]Area Under the Curve (AUC) measures the overall performance of the SEARCH indication algorithm for classifying screening colonoscopy exams according to each viewpoint
[b]Sensitivity for the SEARCH algorithm was fixed at the sensitivity estimate from the KPNC indication algorithm and the corresponding specificity estimate for the SEARCH algorithm was reported
[c]Sources include: referral or clinic notes from the visit that prompted the referral
[d]Sources include: procedure report or pre-procedure notes
[e]Sources include: referral, clinic notes from the visit that prompted the referral, procedure report, pre-procedure notes, electronic data capture for laboratory-confirmed positive FIT/FOBT or iron-deficiency anemia
