# Supplementary material for: Colonoscopy Indication Algorithm Performance Across Diverse Health Care Systems in the PROSPR Consortium
Source: EGEMS (Wash DC). 2019 Aug 2;7(1):37. doi: 10.5334/egems.296 (PMC6676916; doi:10.5334/egems.296)
Supplement: Supplemental Table 2. — Sensitivity and specificity of the KPNC algorithm for classifying diagnostic and surveillance colonoscopy exams, by PROPSR Research Center and source of indication assessment. [file egems-7-1-296-s6.pdf]

Supplemental Table 2. Sensitivity and specificity of the KPNC algorithm for classifying diagnostic and surveillance colonoscopy exams, by PROPSR Research Center and source of indication assessment

	Diagnostic		Surveillance	
	Sensitivity (95% CI)	Specificity (95% CI)	Sensitivity (95% CI)	Specificity (95% CI)
**KPWA**				
Referral[a]	0.91 (0.87,0.95)	0.79 (0.74,0.83)	0.66 (0.56,0.76)	0.92 (0.90,0.95)
Procedure Report/Pre-procedure note[b]	0.89 (0.85,0.94)	0.70 (0.65,0.74)	0.59 (0.50,0.67)	0.94 (0.92,0.96)
Gold Standard Sources[c]	0.89 (0.85,0.93)	0.82 (0.78,0.86)	0.67 (0.59,0.75)	0.96 (0.94,0.98)
**KPNC/SC**				
Referral[a]	0.83 (0.79,0.88)	0.72 (0.67,0.76)	0.60 (0.49,0.71)	0.93 (0.90,0.95)
Procedure Report/Pre-procedure note[b]	0.86 (0.82,0.91)	0.68 (0.64,0.73)	0.51 (0.43,0.60)	0.96 (0.94,0.97)
Gold Standard Sources[c]	0.83 (0.79,0.87)	0.78 (0.74,0.83)	0.59 (0.50,0.69)	0.95 (0.93,0.97)
**Parkland-UTSW**				
Referral[a]	0.87 (0.82,0.91)	0.71 (0.66,0.76)	0.87 (0.77,0.98)	0.5 (0.45,0.54)
Procedure Report/Pre-procedure note[b]	0.79 (0.74,0.85)	0.64 (0.59,0.69)	0.08 (0.01,0.16)	1.00 (1.00,1.00)
Gold Standard Sources[c]	0.78 (0.73,0.82)	0.74 (0.69,0.79)	0.11 (0.01,0.20)	1.00 (1.00,1.00)

[a]Sources include: referral or clinic notes from the visit that prompted the referral
[b]Sources include: procedure report or pre-procedure notes
[c]Sources include: referral, clinic notes from the visit that prompted the referral, procedure report, pre-procedure notes, electronic data capture for laboratory-confirmed positive FIT/FOBT or iron-deficiency anemia
